# Supplementary material for: Occurrence and backtracking of microplastic mass loads including tire wear particles in northern Atlantic air
Source: Nat Commun. 2023 Jun 22;14:3707. doi: 10.1038/s41467-023-39340-5 (PMC10287736; doi:10.1038/s41467-023-39340-5)
Supplement: Supplementary file 2 — Supplementary Information [file 41467_2023_39340_MOESM2_ESM.pdf]

Supplementary Information

Occurrence and backtracking of microplastic mass loads including tire wear particles in northern Atlantic air

Isabel Goßmann[1], Dorte Herzke[2,3], Andreas Held[4], Janina Schulz[1], Vladimir Nikiforov[2] Christoph Georgi[4], Nikolaos Evangeliou[5], Sabine Eckhardt[5], Gunnar Gerdts[6], Oliver Wurl[7], Barbara M. Scholz-Böttcher[1*]

[1]Institute for Chemistry and Biology of the Marine Environment (ICBM), Carl von Ossietzky University of Oldenburg, P.O. Box 2503, 26111 Oldenburg, Germany

[2]NILU - Norwegian Institute for Air Research, The FRAM Centre, P.O. Box 6606, Langnes, 9296 Tromsø, Norway

[3]NIPH – Norwegian Institute for Public Health, P.O.Box 222 Skøyen, 0213 Oslo, Norway

[4]Chair of Environmental Chemistry and Air Research, Technische Universität Berlin, 10623 Berlin, Germany

[5]NILU - Norwegian Institute for Air Research, Instituttveien 18, 2007, Kjeller, Norway

[6]Alfred Wegener Institute, Helmholtz Center for Polar and Marine Research, 27483 Heligoland, Germany

[7]Center for Marine Sensors, Institute for Chemistry and Biology of the Marine Environment (ICBM), Carl von Ossietzky University of Oldenburg, 26382 Wilhelmshaven, Germany

Table S1. Summary of marine atmospheric MP data, acquired by active sampling published according to Allen et al., 2022[1] and further literature.

Reference	Location	MP count [particles m^{-3}]	Size range[b]	Pre-dominant size range[b]	Type of analysis	Polymer composition
Ding et al., 2021[2]	South China Sea	0.013 – 0.063	50 µm - 2.21 mm	<200 µm	visual, FTIR	Polyester (29%), Rayon (19%), PP (15%), PE (13%), PS (10%)
Ding et al., 2022[3]	Northwestern Pacific Ocean	0.0046 – 0.064	10 – 4556 µm		µ-FTIR	Rayon (67%), PET (23%)
Ferrero et al., 2022[4]	Baltic Sea and Gotland Island	0 – 85 (301[a])	? µm – 5 mm	127 µm length 17 µm width	visual, µ-Raman, FTIR	Polyester (39.5%), PC (35.5%), PE (11.8%), PET (5.3%), PU (5.3%)
Liu et al., 2019[5]	Western Pacific Ocean (Shanghai – Mariana Islands)	0 – 1.37	20 µm – 2 mm	318 µm	visual, FTIR	PET (56%), epoxy resin (10%), PE-PP (7%), PS (6%)
Trainic et al., 2020[6]	North Atlantic Ocean	0 – 0.079	5 µm – 5 mm	5 – 10 µm	µ-Raman and visual (confocal microscope)	PS > PE & PP
Wang et al., 2020[7]	Pearl River Estuary, South China Sea, Indian Ocean	0 – 0.077	58 – 2252 µm	851 µm	visual, FTIR	PET (50.0%), PP (22.2%), other (e.g. phenoxy resin, poly(acrylonitrile-co-acrylic acid), poly(ethylene-co propylene) (27.8%)
Wang et al., 2021[8]	South China Sea	0 – 0.013	19 – 948 µm	<200 µm	visual, FTIR	PET (54.55%), PMMA (13.64%), EVA (9.09%), PE (9.09%)

[a]max. value detected in the study, with origin from Gdansk Harbour, Poland; [b]Note that in FTIR and Raman spectroscopy studies, MP sizes are typically reported as length in the largest dimension which is different from the concept of the aerodynamic diameter used when sampling PM2.5 and PM10 fractions. In order to characterize the MP aerodynamic behavior, both the major and the minor dimensions of the particle are important (e.g. Gonda & Abd El Khalik, 1985[9]).

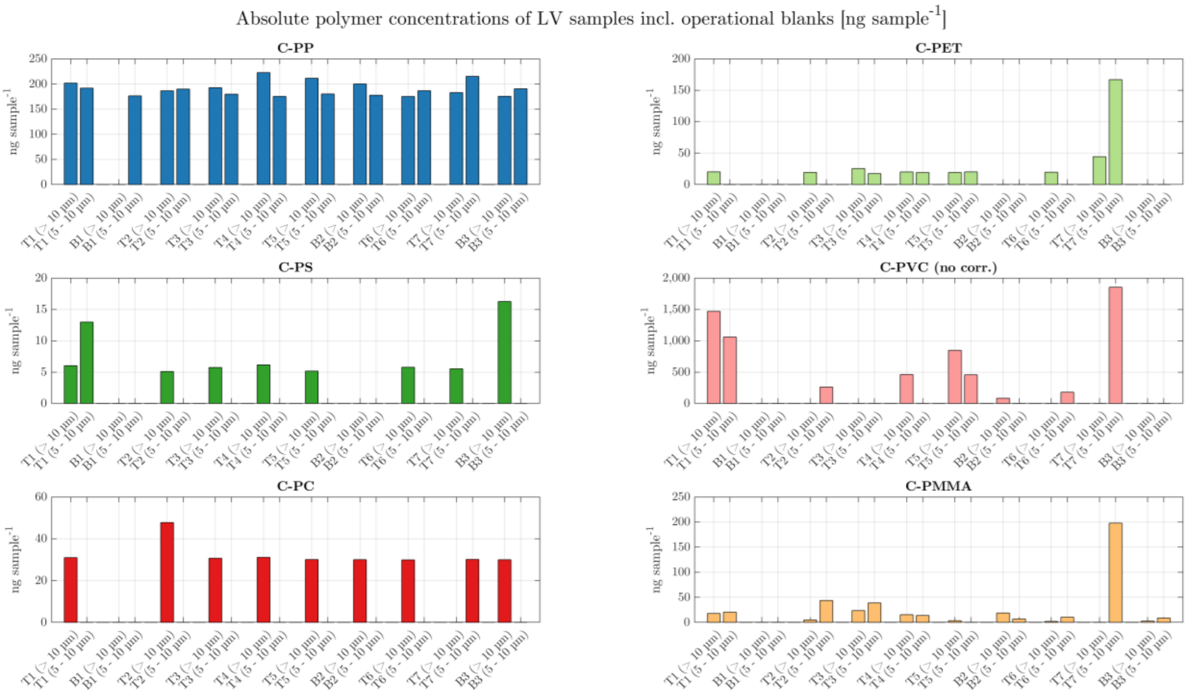

Fig. S1. Absolute polymer concentration ng sample^{-1} without consideration of total sample volume of the low-volume (LV) samples for transects (T1 – T7) and blanks (B1 – B3). The x-axis reflects the chronological order of sample and blank collection.

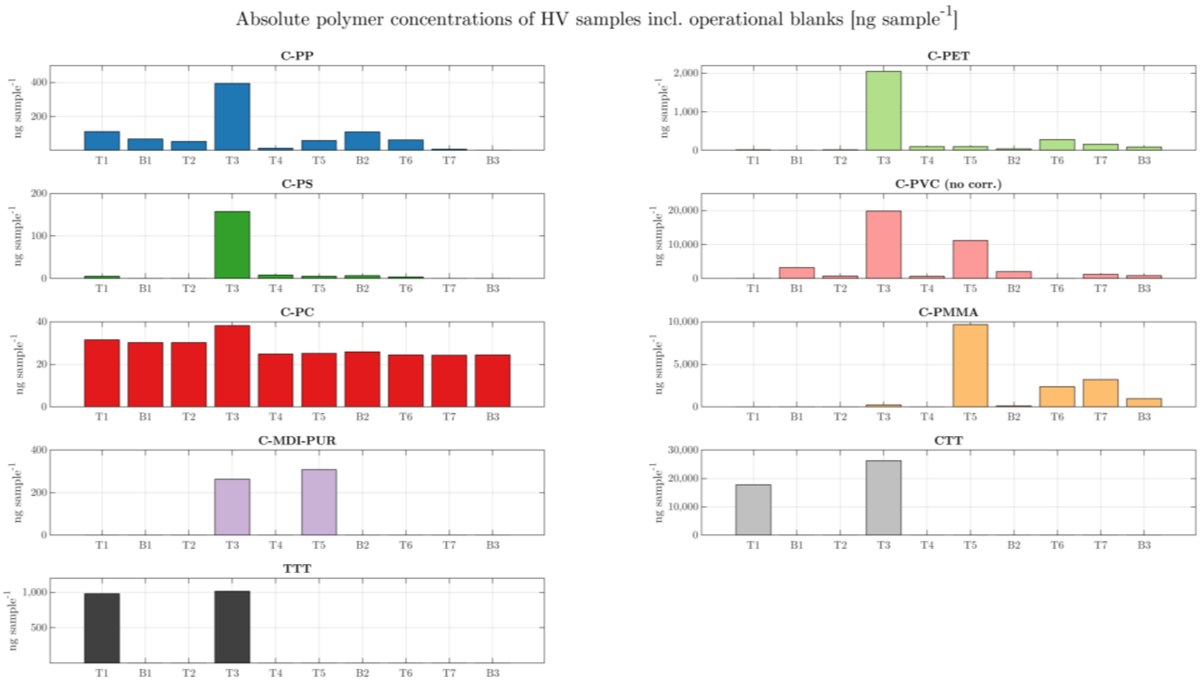

Fig. S2. Absolute polymer concentration ng sample^{-1} without consideration of total sample volume of the high-volume (HV) samples for transects (T1 – T7) and blanks (B1 – B3). The x-axis reflects the chronological order of sample and blank collection.

Table S2. Limit of detection (LOD) and limit of quantification (LOQ) for respective polymers analysed in this study (*representative for a system in optimum condition).

**Polymer**	**Limit of detection (LOD) S/N ≥3 (ng absolute*)**	**Limit of quantification (LOQ) S/N ≥10 (ng absolute*)**
C-PE	100-300	100-300
C-PP	< 100[b]	500
C-PET[a]	10-50	50
C-PS[a]	1	2
C-PVC[a]	10-50	50
C-PMMA	< 100[b]	100-300
C-PC[a]	1	2
C-MDI-PUR	100-300[b]	500
C-PA6	100-300[b]	500

[a]based on dissolved standards; [b]derived from lowest calibration point for orientation only

Table S3. Quantitative results of the low-volume (LV) samplers in ng sample^{-1} for the 5 -10 µm and > 10 µm size fractions. C-PE and C-PA6 are excluded from the table since they were not detected in any transect nor sampler type.

	C-PP	C-PET	C-PS	C-PVC	C-PC	C-PMMA	C-MDI-PUR	CTT	TTT
**5 – 10 µm**	**ng sample^{-1}**								
**T1**	191.8	n.d.	13.0	1,059.9	n.q.	20.4	n.d.	n.d.	n.d.
**B1**	176.4	n.d.	n.d.	0.0	n.q.	n.q.	n.d.	n.d.	n.d.
**T2**	189.8	n.d.	n.d.	261.4	n.q.	43.6	n.d.	n.d.	n.d.
**T3**	179.8	17.4	n.d.	n.q.	n.q.	38.7	n.d.	n.d.	n.d.
**T4**	175.3	19.1	n.d.	n.q.	n.q.	13.7	n.d.	n.d.	n.d.
**T5**	180.2	20.2	n.d.	456.7	n.q.	n.q.	n.d.	n.d.	n.d.
**B2**	177.7	n.d.	n.d.	n.q.	n.q.	6.9	n.d.	n.d.	n.d.
**T6**	186.6	n.d.	n.d.	179.6	n.q.	10.4	n.d.	n.d.	n.d.
**T7**	215.2	166.9	n.d.	1,853.0	30.1	197.8	n.d.	n.d.	n.d.
**B3**	190.5	n.d.	n.d.	n.q.	n.q.	8.6	n.d.	n.d.	n.d.
**> 10 µm**	**ng sample^{-1}**								
**T1**	201.7	20.2	6.0	1,468.4	31.0	17.7	n.d.	n.d.	n.d.
**B1**	Not analysed, filter fell down								
**T2**	186.4	19.3	5.1	0.1	47.8	4.9	n.d.	n.d.	n.d.
**T3**	192.7	25.6	5.7	n.q.	30.7	23.4	n.d.	n.d.	n.d.
**T4**	222.6	20.0	6.2	461.9	31.1	15.3	n.d.	n.d.	n.d.
**T5**	211.4	19.3	5.2	847.6	30.0	3.4	n.d.	n.d.	n.d.
**B2**	200.2	n.d.	n.d.	83.5	30.0	18.7	n.d.	n.d.	n.d.
**T6**	175.2	19.7	5.8	0.2	29.9	1.9	n.d.	n.d.	n.d.
**T7**	182.9	44.3	5.5	n.q.	n.q.	n.q.	n.d.	n.d.	n.d.
**B3**	175.6	n.d.	16.3	n.q.	30.0	2.4	n.d.	n.d.	n.d.

n.d. = not detectable; n.q. = not quantifiable

Table S4. Quantitative results of the high-volume (HV) samplers KO and VM and the mean values of the transects T (bold) in ng sample^{-1} for the > 10 µm size fractions. C-PE and C-PA6 are excluded from the table since they were not detected in any transect nor sampler type.

	**C-PP**	**C-PET**	**C-PS**	**C-PVC**	**C-PC**	**C-PMMA**	**C-MDI-PUR**	**CTT**	**TTT**
	**ng sample^{-1}**								
KO1	n.q.	18.2	8.3	n.q.	32.6	n.q.	n.d.	17,773.8	991.7
VM1	224.5	27.0	1.8	n.q.	30.5	n.q.	n.d.	17,807.6	969.9
**T1**	**112.2**	**22.6**	**5.1**	**n.q.**	**31.5**	**n.q.**	**n.d.**	**17,790.7**	**980.8**
B1	n.q.	67.8	n.q.	n.q.	30.3	30.3	n.d.	n.d.	n.d.
KO2	39.9	18.0	n.q.	1091.4	30.0	n.q.	n.d.	n.d.	n.d.
VM2	65.6	20.7	0.4	453.8	30.3	n.q.	n.d.	n.d.	n.d.
**T2**	**52.7**	**19.4**	**0.2**	**772.6**	**30.2**	**n.q.**	**n.d.**	**n.d.**	**n.d.**
KO3	449.1	3290.5	280.8	39,523.1	49.0	501.4	525.7	33,164.3	1,069.1
VM3	339.5	793.9	34.9	n.q.	27.3	n.q.	n.d.	19,360.7	963.5
**T3**	**394.3**	**2,042.2**	**157.9**	**19,761.6**	**38.1**	**250.7**	**262.9**	**26,262.5**	**1,016.3**
KO4	23.0	90.2	7.6	661.7	24.8	n.q.	n.d.	n.d.	n.d.
VM4	4.7	105.2	8.6	724.8	24.8	n.q.	n.d.	n.d.	n.d.
**T4**	**13.8**	**97.7**	**8.1**	**693.3**	**24.8**	**n.q.**	**n.d.**	**n.d.**	**n.d.**
KO5	59.9	119.0	n.q.	21680.4	24.7	19,290.9	617.2	n.d.	n.d.
VM5	56.9	79.9	10.7	702.1	25.5	n.q.	n.d.	n.d.	n.d.
**T5**	**58.4**	**99.5**	**5.3**	**1,1191.3**	**25.1**	**9,645.4**	**308.6**	**n.d.**	**n.d.**
B2	110.2	45.7	6.6	2,057.5	25.9	121.2	n.d.	n.d.	n.d.
KO6	13.2	108.1	0.0	n.q.	24.3	2064.0	n.d.	n.d.	n.d.
VM6	111.5	451.4	7.5	n.q.	24.6	2694.3	n.d.	n.d.	n.d.
**T6**	**62.4**	**279.8**	**3.7**	**n.q.**	**24.5**	**2,379.2**	**n.d.**	**n.d.**	**n.d.**
KO7	0.0	104.0	n.q.	607.4	24.4	1,904.3	n.d.	n.d.	n.d.
VM7	14.6	219.1	n.q.	1,896.4	24.3	4,541.2	n.d.	n.d.	n.d.
**T7**	**7.3**	**161.5**	**n.q.**	**1,251.9**	**24.3**	**3,222.7**	**n.d.**	**n.d.**	**n.d.**
B3	n.q.	89.8	n.q.	885.8	24.5	958.8	n.d.	n.d.	n.d.

n.d. = not detectable; n.q. = not quantifiable

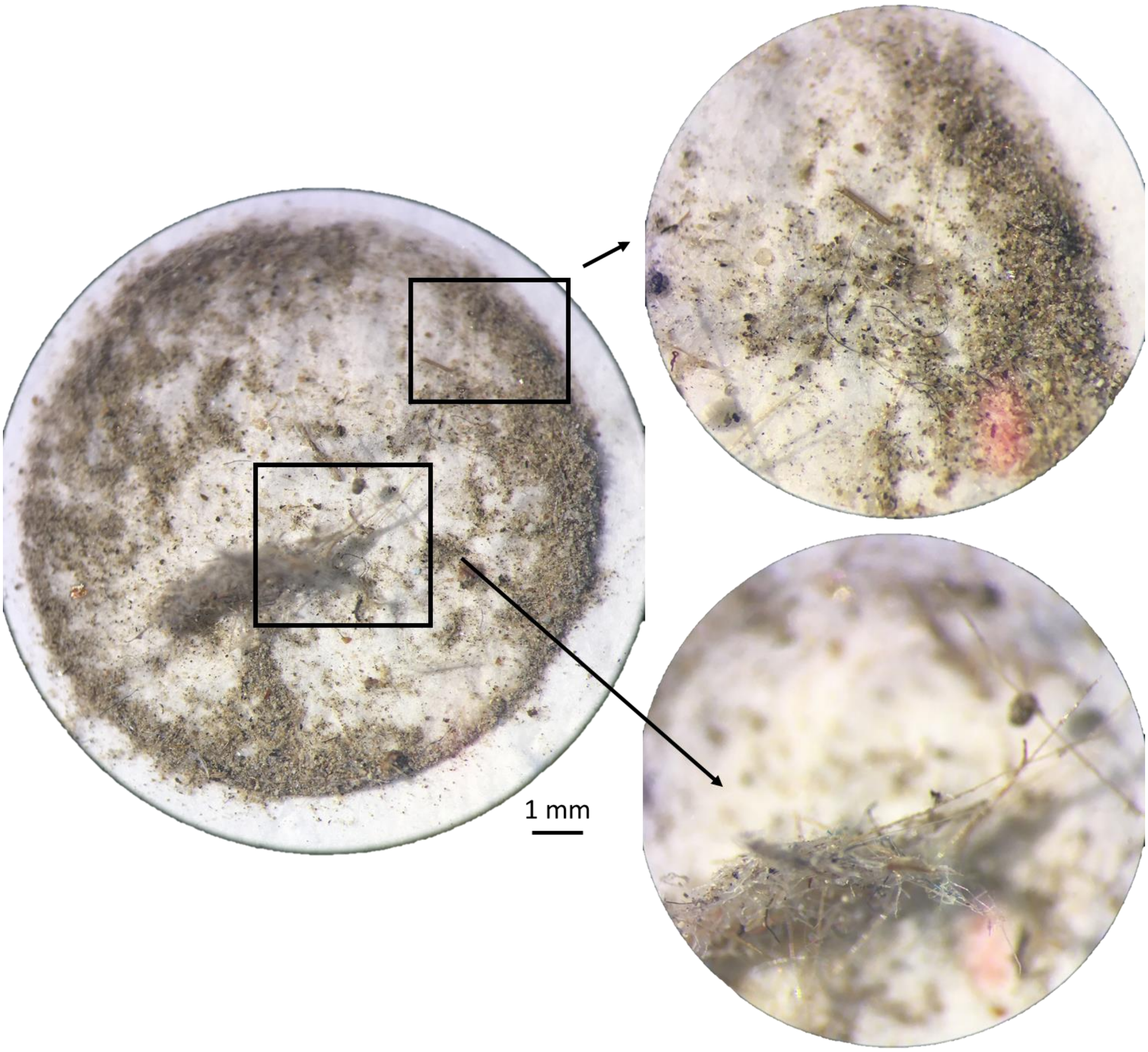

Fig. S3. Filter cake of HV sample KO3 from transect T3 with clearly visible fiber accumulation.

Atmospheric transport and dispersion models

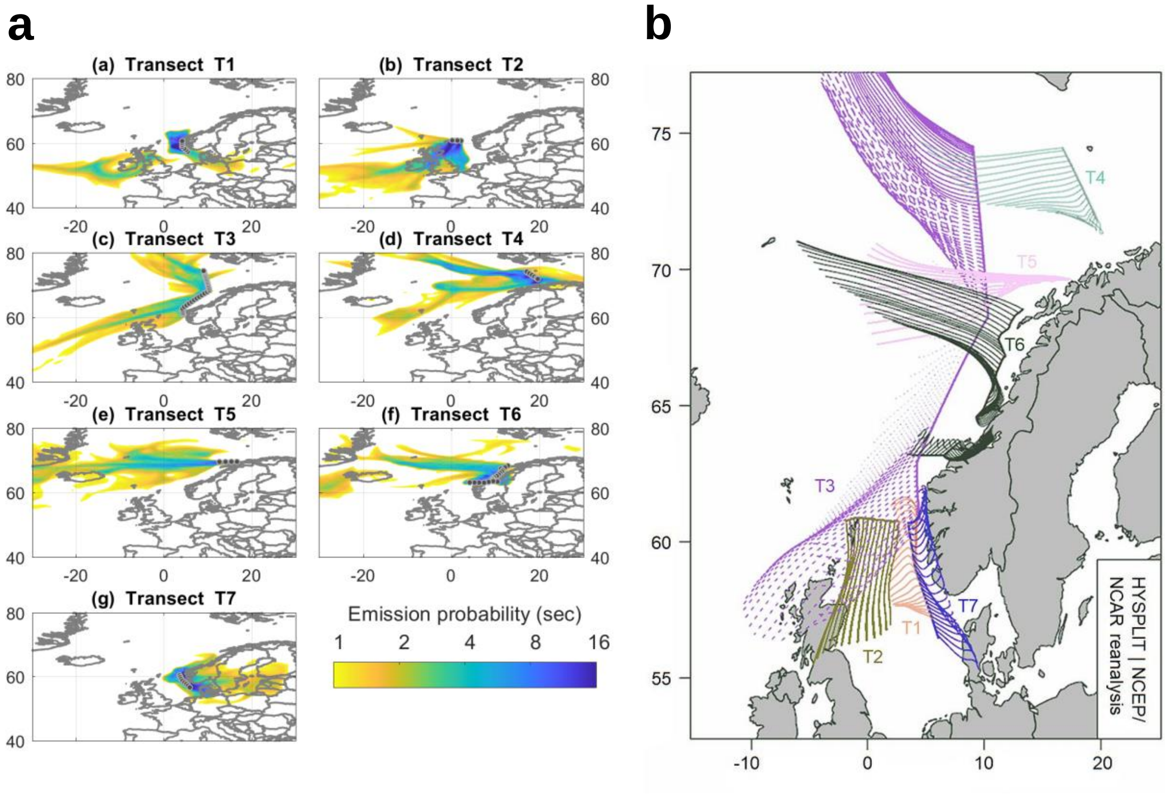

Fig. S4. FLEXPART FLEXible PARTicle dispersion model) and HYSPLIT (Hybrid Single-Particle Lagrangian Integrated Trajectory) model results for evaluation of particle and air mass origin for the respective transects, T1 - T7. (a) FLEXPART footprints simulating emissions of microplastic (MP) < 10 µm size fraction at heights from 0 – 100 m above sea level for a duration of 30 days. (b) HYSPLIT back trajectories for the height of 30 m above sea level for a 24-hour duration.

Table S5. Sampling details for the low-volume (LV) sampling.

Transect	start (in UTC)	End (in UTC)	Start (coordinates)	End (coordinates)	Volume air [m³]
**T1**	2021-06-06 08:00:00	2021-06-07 04:40:00	57° 11,705' N 005° 31,688' E	60° 44,967' N 004° 01,033' E	92.5
**B1**	2021-06-06 08:00:00	2021-06-07 04:40:00	57° 11,705' N 005° 31,688' E	60° 44,967' N 004° 01,033' E	
**T2**	2021-06-08 18:00:00	2021-06-09 06:00:00	60° 45,016' N 002° 38,951' E	60° 46,176' N 001° 49,459' W	53.6
**T3**	2021-06-13 11:00:00	2021-06-17 06:00:00	60° 46,847' N 004° 36,204' E	74° 31,340' N 008° 59,414' E	417.0
**T4**	2021-06-20 10:30:00	2021-06-21 04:00:00	74° 26,573' N 016° 44,146' E	71° 50,691' N 019° 38,382' E	78.9
**B2**	2021-06-25 17:09:00	2021-06-25 17:10:00	69° 38,792‘ N 017° 17,684‘ E	69° 38,792‘ N 017° 17,684‘ E	
**T5**	2021-06-25 17:10:00	2021-06-26 05:25:00	69° 38,792‘ N 017° 17,684‘ E	69° 30,100‘ N 011° 47,634‘ E	55.6
**T6**	2021-06-29 12:00:00	2021-07-02 03:50:00	68° 33,017‘ N 013° 24,097‘ E	63° 11,532‘ N 003° 22,853‘ E	164.4
**T7**	2021-07-04 07:50:00	2021-07-05 07:50:00	60° 41,173‘ N 003° 27,712‘ E	56° 26,053‘ N 006° 04,095‘ E	108.1
**B3**	2021-07-05 07:50:00	2021-07-05 07:51:00	56° 26,053‘ N 006° 04,095‘ E	56° 26,053‘ N 006° 04,095‘ E	

Table S6. Sampling details for the high-volume (HV) sampling.

Transect	Sampler	start (in UTC)	End (in UTC)	Start (coord.)	End (coord.)	Volume air [m³]
**T1**	VM1	2021-06-06 08:00:00	2021-06-07 05:00:00	57° 11,705' N 005° 31,688' E	60° 44,967' N 004° 01,033' E	504
	KO1	2021-06-06 08:00:00	2021-06-07 05:00:00	57° 11,705' N 005° 31,688' E	60° 44,967' N 004° 01,033' E	504
**B1**		2021-06-07 05:05:00	2021-06-07 05:06:00	60° 44,967' N 004° 01,033' E	60° 44,967' N 004° 01,033' E	
**T2**	VM2	2021-06-08 18:00:00	2021-06-09 06:00:00	60° 45,016' N 002° 38,951' E	60° 46,176' N 001° 49,459' W	288
	KO2	2021-06-08 18:00:00	2021-06-09 06:00:00	60° 45,016' N 002° 38,951' E	60° 46,176' N 001° 49,459' W	288
**T3[a]**	VM3a	2021-06-13 11:00:00	2021-06-14 11:00:00	60° 46,847' N 004° 36,204' E	64° 39,213' N 006° 02,122' E	1,728
	VM3b	2021-06-14 11:00:00	2021-06-15 11:00:00	64° 39,213' N 006° 02,122' E	68° 40,381' N 010° 16,242' E	
	VM3c	2021-06-15 11:00:00	2021-06-16 11:00:00	68° 40,381' N 010° 16,242' E	72° 23,252' N 009° 30,462' E	
	KO3	2021-06-13 11:00:00	2021-06-17 06:00:00	60° 46,847' N 004° 36,204' E	74° 31,340' N 008° 59,414' E	2,184

**T4**	VM4	2021-06-20 10:30:00	2021-06-21 03:30:00	74° 26,573' N 016° 44,146' E	71° 50,691' N 019° 38,382' E	408
	KO4	2021-06-20 10:30:00	2021-06-21 03:30:00	74° 26,573' N 016° 44,146' E	71° 50,691' N 019° 38,382' E	408
**B2**		2021-06-25 17:09:00	2021-06-25 17:10:00	69° 38,792‘ N 017° 17,684‘ E	69° 38,792‘ N 017° 17,684‘ E	
**T5**	VM5	2021-06-25 17:10:00	2021-06-26 05:10:00	69° 38,792‘ N 017° 17,684‘ E	69° 30,100‘ N 011° 47,634‘ E	288
	KO5	2021-06-25 17:10:00	2021-06-26 05:10:00	69° 38,792‘ N 017° 17,684‘ E	69° 30,100‘ N 011° 47,634‘ E	288
**T6**	VM6	2021-06-29 12:00:00	2021-07-02 03:50:00	68° 33,017‘ N 013° 24,097‘ E	63° 11,532‘ N 003° 22,853‘ E	1,532
	KO6	2021-06-29 12:00:00	2021-07-02 03:50:00	68° 33,017‘ N 013° 24,097‘ E	63° 11,532‘ N 003° 22,853‘ E	1,532
**T7**	VM7	2021-07-04 07:50:00	2021-07-05 07:50:00	60° 41,173‘ N 003° 27,712‘ E	56° 26,053‘ N 006° 04,095‘ E	576
	KO7	2021-07-04 07:50:00	2021-07-05 07:50:00	60° 41,173‘ N 003° 27,712‘ E	56° 26,053‘ N 006° 04,095‘ E	576
**B3**		2021-07-05 07:50:00	2021-07-05 07:51:00	56° 26,053‘ N 006° 04,095‘ E	56° 26,053‘ N 006° 04,095‘ E	

[a]Due to technical problems of HV air sampler VM during transect T3, the pre-cleaned aluminum rings were changed two times during sampling. For MP quantification, the lab-blank corrected raw data of the three sub-samples were added up.

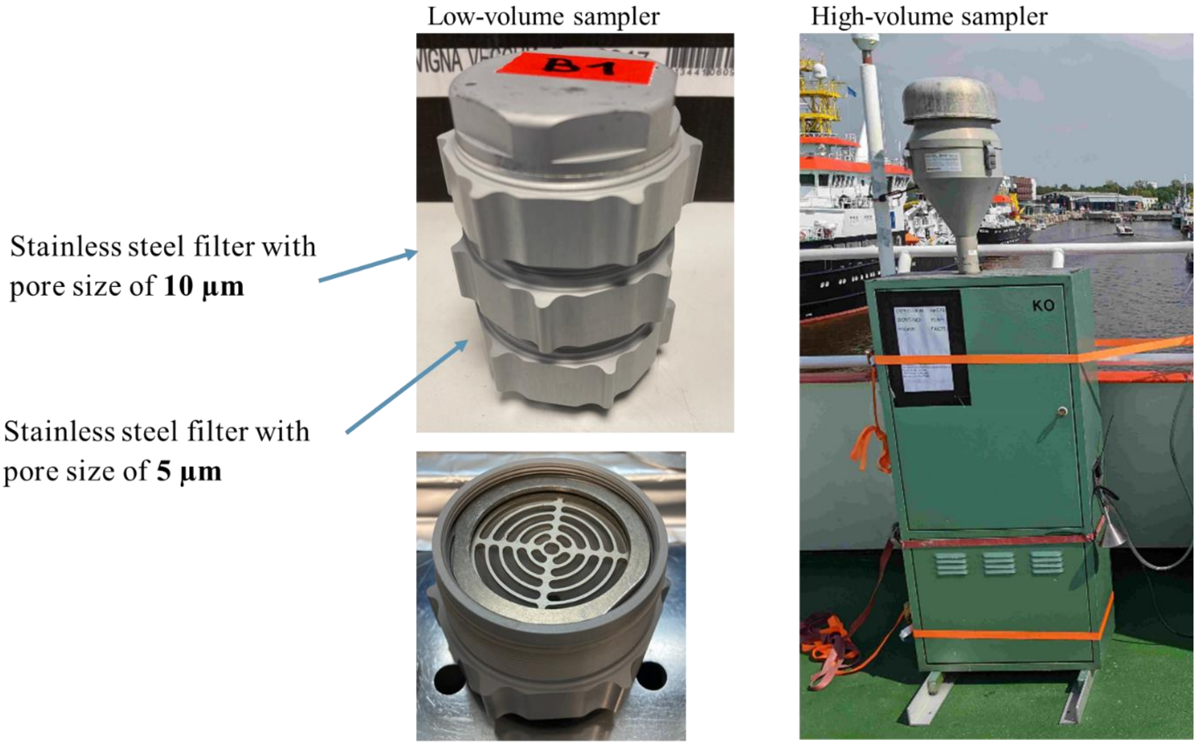

Fig. S5. Pictures of the low- and high-volume sampler.

Table S7. Conditions for Py-GC/MS.

**Micro furnace pyrolyzer & autosampler (EGA/PY-3030D, AS-1020E, (FrontierLabs))**	
Carrier gas	Helium
Temperature	590°C
Pyrolysis time	1 min
Transfer line temperature	320°C
**Gas chromatograph (6890N (Agilent))**	
Injector	Split/split less
Mode	Split 1:12.5
Temperature	300°C
Pre-column	Trajan P/N 064062; 10 m x 250 µm/363 µm VSDP tubing
Column	DB5 (J&W); 30 m x 0.25 mm ID, film thickness 0.25 µm
Flow (const.)	1.2 mL min^{-1}
Temperature program	35°C (2 min) → 310°C (30 min) at 4°C min^{-1} → hold 60 min
Transfer line temperature	280°C
**Mass spectrometer (MSD 5973 (Agilent)**	
Ionization energy	70 eV
Scan rate	2.48 scans s^{-1}
Scan range	*m/z* 50-650
EI-Source temperature	230°C
Quadrupole temperature	150°C

Table S8. Table of characteristic Py-GC/MS composition products used for identification and quantification of detected polymer clusters indicated by “C-“ as pure polymers and potentially included polymer related derivatives according to Goßmann et al., 2022[10].

Basic polymer cluster	Cluster associated compounds	Characteristic decomposition products	Indicator ions [*m/z*]
**C-PE**	HDPE, LDPE, PE-containing copolymers and rubbers, ethylene-vinyl acetate (EVA), EPDM rubber	Alkanes (e.g. C20)	282 [M], 85
		α-Alkanes (e.g. C20)	280 [M], 83
		**α,ω-Alkanes[a]** (e.g. C20)	278 [M], 95, **82**
**C-PP**	PP, EPDM rubber	**2,4-Dimethylhept-1-ene**	126 [M], **70**
		2,4,6,8-Tetramethyl-1-undecene[b]	210 [M], 100, 69
		2,4,6,8-Tetramethyl-1-undecene[c]	210 [M], 100, 69
		2,4,6,8-Tetramethyl-1-undecene[d]	210 [M], 100, 69
**C-PS**	PS, PS-containing copolymers (e.g., ABS, SAN), PS- or acryl styrene binders, varnish	Styrene	104 [M]
		2,4-Diphenyl-1-butene	208 [M], 91
		**2,4,6-Triphenyl-1-hexene**	312 [M], **91**
**C-PVC**	PVC (hard and plasticized), chlorinated rubber	Benzene	78 [M]
		Chlorbenzene	112 [M]
		**Napthalene**	**128** [M]
**C-PET**	PET, polybutylene terephthalate	**Dimethyl terephthalate[e]**	194 [M], **163**
**C-PMMA**	PMMA; polyalkylated methacrylate, acryl containing binder	Methacrylate	86 [M], 55
		**Methyl methacrylate**	**10**0 [M], 69
**C-PC**	PC, epoxide resin	*p*-Methoxy-*tert*-butylbenzene[e]	242 [M], 164, 149
		**2,2-Bis(4’-methoxy-phenyl)propane[e]**	256 [M], **241**
**C-PA6**	PA6	**ε-Caprolactam**	**11**3 [M]
		**N-methyl caprolactam[e]**	**127** [M]
**C-MDI-PUR**	MDI-PUR, MDI-PUR based formulations	4,4’-Methylenbis(N-methylaniline)[e]	226 [M]
		N,N-Dimethyl-4-(4-methylamino)benzylanilin[e]	240 [M]
		**4,4’-Methylenbis(N,N-dimethylaniline)[e]**	**254** [M], 253, 210, 134
**TTT**	Truck tire tread, bus tire tread	**2,4-Dimethyl-4-vinylcyclohexene (DMVCH)**	136 [M], 121, 93, **68**
		1-Methyl-4-(1-methylethenyl)-cyclohexene	136 [M], 121, 93, 68
**CTT**	Car tire tread	Ethenylbenzene	104 [M], 78, 51
		**Cyclohexenylbenzene (SB)**	158 [M], 129, 115, **104**

[m/z] = mass to charge ratio; [M] = molecular ion; bold = indicator ions used for calibration and quantification; [a]Mean of *n*-C_{16}-C_{26}-alkadiens used for quantification of PE. [b]isotactic. [c]heterotactic. [d]syndiotactic. [e]only after TMAH treatment.

Table S9. Plastic standards used for quantification.

Polymer standard	Acronym	Additional information	Supplier
Polyamide 6 (K891), Alkulon® K222-D	PA6	Low viscosity	Ter Hell, GmbH, Hamburg, Germany
Polycarbonate, Markoplon 2558	PC		Bayer Material, Science
Polyethylene, Lupolen 4261 AG UV	HDPE	High density	LyondellBasell
Polyethylene terephthalate, NEOPET 80	PET		Neogroup
Polymethyl methacrylate, PLEXIGLAS® 7N	PMMA		Plexiglas®
Polypropylene, HL508FB	PP		Borealis
Polystyrene, TOTAL PS impact 7240	PS	High impact PS for extrusion industry	Ter Hell, GmbH, Hamburg, Germany
Polystyrene, Styrolution PS 158N/L	PS	Raw material	IINEOS Styrosolution
Polyurethane	PUR	MDI-PUR	GEBA GmbH
Polyvinylchloride, Vinnolit S3268	PVC	Hard PVC, raw material	Vinnolit
All-season truck tire tread	TTT		Goodyear Tire & Rubber Company
All-season car tire tread	CTT		Semperit (Continental AG)

Table S10. Overview of calibration measurements.

	PE	PP	PET	PS	PVC	PC	PMMA	PA6	MDI-PUR	CTT	TTT
**Low-volume samples**											
Date of measurement sequence: **21.02.22**											
**B**	/	-0.05332	-0.03803	-0.00436	0.01165	-0.24395	0.37917	/	-0.18625	/	/
**Slope**	/	0.32666	2.40678	1.18516	0.04308	8.1797	0.32929	/	0.12031	/	/
**r²**	/	0.92801	0.98071	0.79199	0.91577	0.96756	0.9581	/	0.79199	/	/
**High-volume samples**											
Date of measurement sequence: **21.02.22**											
**B**	/	+	-0.03803	0.00236	0.01304	-0.24395	0.29926	/	-0.17117	-0.06598	-0.19719
**Slope**	/	+	2.40678	0.80346	0.04212	8.1797	0.33749	/	0.12545	0.0039	0.20466
**r²**	/	+	0.98071	0.98299	0.81325	0.96756	0.92302	/	0.84494	0.85046	0.85684
Date of measurement sequence: **03.03.22**											
**B**	/	+	-0.0867	-0.00488	0.0096	-0.21184	0.08916	/	-0.01688	-0.06627	-0.19719
**Slope**	/	+	2.61034	0.99491	0.04102	8.67124	0.61426	/	0.05375	0.0039	0.20466
**r²**	/	+	0.92496	0.92572	0.91309	0.98733	0.96461	/	0.86592	0.92807	0.85684
Date of measurement sequence: **28.03.22**											
**B**	/	+	-0.0852	0.01204	0.02129	-0.16804	0.06868	/	0.02272	/	/
**Slope**	/	+	1.54062	0.92369	0.04332	6.95696	0.57043	/	0.03838	/	/
**r²**	/	+	0.67752	0.94112	0.82014	0.99002	0.97171	/	0.66994	/	/

B = y-intercept, r^2 = coefficient of determination, / = not identified in samples and therefore not calibrated, + = 1-Point-calibration

Supplementary References

1. Allen, D. *et al.* Microplastics and nanoplastics in the marine-atmosphere environment. *Nat. Rev. Earth Environ.* **3**, 393–405 (2022).

2. Ding, Y. *et al.* The abundance and characteristics of atmospheric microplastic deposition in the northwestern South China Sea in the fall. *Atmos. Environ.* **253**, 118389 (2021).

3. Ding, J. *et al.* Atmospheric microplastics in the Northwestern Pacific Ocean: Distribution, source, and deposition. *Sci. Total Environ.* **829**, 154337 (2022).

4. Ferrero, L. *et al.* Airborne and marine microplastics from an oceanographic survey at the Baltic Sea: An emerging role of air-sea interaction? *Sci. Total Environ.* **824**, 153709 (2022).

5. Liu, K. *et al.* Consistent Transport of Terrestrial Microplastics to the Ocean through Atmosphere. *Environ. Sci. Technol.* **53**, 10612–10619 (2019).

6. Trainic, M. *et al.* Airborne microplastic particles detected in the remote marine atmosphere. *Commun. Earth Environ.* **1**, 64 (2020).

7. Wang, X. *et al.* Atmospheric microplastic over the South China Sea and East Indian

Ocean: abundance, distribution and source. *J. Hazard. Mater.* **389**, 121846 (2020).

8. Wang, X. *et al.* Efficient transport of atmospheric microplastics onto the continent via the East Asian summer monsoon. *J. Hazard. Mater.* **414**, 125477 (2021).

9. Gonda, I. & Abd El Khalik, A. F. On the Calculation of Aerodynamic Diameters of Fibers. *Aerosol Sci. Technol.* **4**, 233–238 (1985).

10. Goßmann, I., Süßmuth, R. & Scholz-Böttcher, B. M. Plastic in the air?! - Spider webs as spatial and temporal mirror for microplastics including tire wear particles in urban air. *Sci. Total Environ.* **832**, 155008 (2022).
